# Nutritional Management of Medical Inpatients

**DOI:** 10.3390/jcm8081130

**Published:** 2019-07-30

**Authors:** Emilie Reber, Filomena Gomes, Lia Bally, Philipp Schuetz, Zeno Stanga

**Affiliations:** 1 Department of Diabetes, Endocrinology, Nutritional Medicine and Metabolism, Bern University Hospital, and University of Bern, 3010 Bern, Switzerland; 2 The New York Academy of Sciences, New York, NY 10007, USA; 3 Department of Medical University, Division of General Internal and Emergency Medicine, Kantonsspital Aarau, 5000 Aarau, Switzerland; 4 Department for Clinical Research, Medical Faculty, University of Basel, 4001 Basel, Switzerland

**Keywords:** malnutrition, nutritional management, nutritional therapy

## Abstract

Malnutrition is a common condition in hospitalized patients that is often underdiagnosed and undertreated. Hospital malnutrition has multifactorial causes and is associated with negative clinical and economic outcomes. There is now growing evidence from clinical trials for the efficiency and efficacy of nutritional support in the medical inpatient population. Since many medical inpatients at nutritional risk or malnourished are polymorbid (i.e., suffer from multiple comorbidities), this makes the provision of adequate nutritional support a challenging task, given that most of the clinical nutrition guidelines are dedicated to single diseases. This review summarizes the current level of evidence for nutritional support in not critically ill polymorbid medical inpatients.

## 1. Introduction

Hippocrates of Kos, one of the most outstanding figures in the history of medicine in the fourth to fifth century BCE stated that “The patient ought likewise to be consider’d, whether he is able to hold out with the prescribed diet, even in the height of the disease; for if the diet is not sufficient, the patient will grow too faint, and be overcome by the disease.” [1]. He considered nutritional interventions to cure diseases as reflected in his well-known statement “Let food be thy medicine and medicine be thy food”.

Around 80 years ago, Studley described weight loss of >20% of body weight as a factor increasing mortality (+33%) in chronic peptic ulcer patients undergoing non-emergency surgery, “regardless of the appearance of the individual” [2]. Some years later, more than 30 years ago, concerns regarding prevalence and adverse effects of disease-related malnutrition (malnutrition triggered by illness or disease) and in hospitalized patients were first reported [3,4,5]. Nowadays, the link between malnutrition and clinical negative outcomes, e.g., muscle wasting, higher infection rates, longer length of hospital stay, morbidity and mortality rates, is clearly established [6,7,8,9,10]. In high-income countries, where access to food should not be limited, every third patient is at risk for malnutrition or already malnourished at hospital admission [11]. Most patients further lose weight during their hospital stay, and, as consequence, their nutritional status deteriorates. A study of Barton et al. showed that 40% of the hospital food served to patients is left on the plates and returned, thus resulting in patients eating less than 80% of their individual energy and protein requirements [12]. There are several factors leading to progressive unintentional weight loss, such as gastrointestinal symptoms, inactivity, depression or low mood, advanced age, effects of illness on protein and energy homeostasis, protein catabolism, inflammation, hormonal function and loss of appetite [13]. Loss of appetite may develop during hospital stays either as a consequence of an underlying disease or treatment or preexist as a primary condition. This loss of appetite arises as a physiological response to acute illness and predisposes inpatients to serious caloric and protein deficits [14]. In combination with immobilization and a pronounced inflammatory and endocrine stress response, these nutritional deficits contribute to muscle wasting and progressive deterioration of metabolic and functional status, particularly in medical inpatients with multiple morbidities [9,15]. An initial loss of appetite does, however, not confer a risk to fail achievement of nutritional targets since this improves quite rapidly after the initiation of nutritional support [16].

There is some evidence from high-quality trials in critical care settings reporting harmful effects of hypercaloric replacement nutrition strategies [14]. These negative effects might be explained by suppression of autophagy with inadequate clearance of acute cell damage associated with illness [17]. Autophagy is a body mechanism to get rid of damaged cells organelles and toxic products. Loss of appetite may therefore be a protective mechanism in an acute disease with the goals to accelerate recovery from disease by improving autophagy. Importantly, in patients suffering from chronic diseases, this protective physiological response may have been exaggerated thereby causing malnutrition. Thus, in patients with multiple chronic diseases, who have milder disease severities, lost weight and muscle mass over time, use of an adequate eucaloric nutritional support has a potentially positive effect. They might have better metabolism and nutrients use due to decreased insulin resistance and decreased risk that nutrition would interfere with autophagy [7,18]. Thus, since critically ill patients have different nutritional needs from the patients on the medical wards, this review focuses on the nutritional support of non-critically ill medical inpatients; as such, the critically ill population is the focus of another publication in this special issue (“Kopp-Lugli et al. Nutrition therapy in critically ill patients treated on intensive and intermediate care units: a literature review”).

Understanding the optimal use of nutritional support is highly complex because timing, route of delivery, and the amount and type of nutrients may all have important roles and potentially affect patient’s outcomes. Furthermore, it has been questioned whether nutritional support in polymorbid medical inpatients differs from the ones suffering from a single disease. Polymorbidity (or multimorbidity) is mostly defined as the co-occurrence of at least two chronic medical conditions in one patient. Polymorbidity is frequent in hospitals and, despite being common in older people, is not necessarily associated with higher age. Nevertheless, older adult patients require special attention, as they tend to be more polymorbid than younger patients are. In addition to the disease burden, older patients often experience malnutrition by multifactor, such as anorexia of aging, presbyphagia and dysphagia due to sarcopenia [19,20,21]. While there are plenty of clinical nutrition guidelines available focusing on individual diseases, [22,23,24,25,26] Gomes et al. recently published new guidelines for the nutritional support in polymorbid medical inpatients [13].

## 2. Today’s Clinical Evidence Level

Recent meta-analyses, which investigated the effects of nutritional support on clinical outcomes concluded that nutritional support leads to an increase in energy and protein intake, an increase in body weight but there were no significant effects on clinical outcomes such as mortality and morbidity, and only little effect on non-elective hospital readmissions [27,28]. The results showed no benefit in critical ill patients, possibly due to a suppression of autophagy and increased risk of refeeding syndrome [28]. In fact, these systematic reviews found (from the included heterogeneous trials and with high risk for bias) an important lack of evidence in this area, as high quality randomized, interventional trials, needed to establish causal relationships, were missing. Thus, the lack of significant results regarding mortality and morbidity may be rather due to methodological issues and low statistical power, and not lack of effectiveness per se.

In fact, other recent studies showed results that are more promising. The multicenter randomized placebo controlled double-blind “NOURISH-trial”, conducted by Deutz and his colleagues, evaluated the effect of specialized, energy-dense ONS on postdischarge outcomes including nonelective readmission and mortality in initially hospitalized, malnourished, older adults. Patients were randomly allocated to receive during hospitalization and after discharge either a specialized, energy-dense ONS or a carbohydrate-only ONS. Results showed that energy-dense, specialized ONS extending beyond discharge improved body weight but could not reduce readmission rate. A significant reduction in 90-day mortality in the intervention group compared to the placebo group (4.8% vs. 9.7%) resulting in a number needed to treat (NNT) of 20 [29]. However, it remains unclear whether this beneficial effect is attributable to the specific formula (containing a leucine metabolite) used in this study or the high protein and energy amount provided by the oral nutritional supplement, given that control patients received a low protein and calorie placebo product. The very recently published open-label, non-blinded, multicenter, randomized-controlled trial, the “EFFORT trial”, conducted by Schuetz and colleagues, assessed the effect of a protocol-guided individualized nutritional support to reach nutritional needs (determined either with the Harris-Benedict formula or with indirect calorimetry) of medical inpatients. Protocol-guided nutritional support reduced the primary outcome (all-cause mortality, admission to intensive care, non-elective hospital readmission, major complications, and decline in functional status) by 4% (22.9% vs. 26.9%) by 30 days when compared to usual care, translating into a NNT of 25 to prevent one severe complication [30]. Additionally, mortality rate was significantly lower in the intervention group compared to the control group (7.2% vs. 9.9%, NNT of 37) and notable improvements in functional outcomes and in quality of life measures were observed. Notably, in the nutrition support group, 91% received oral nutrition, and, perhaps most importantly, an individualized nutritional care plan from a specialist dietitian. Conversely, enteral or parenteral nutrition was used in eight and 12 participants respectively [30]. The effect of nutritional support on the risk for the primary endpoint was consistent across predefined subgroups. Thus, these results provide strong evidence for the concept of systematically screening medical inpatients on hospital admission in terms of nutritional risk, independent of the medical condition, followed by a nutritional assessment and initiation of individualized nutritional support in at-risk patients. The results also contradict the hypothesis that provision of nutritional support during the acute phase of illness would have harmful effects—at least in the non-critically ill setting [30]. Unlike the NOURISH trial that investigated the effect of a specific formula of an oral nutritional supplement, within EFFORT a variety of nutritional support strategies were used by trained dieticians to reach the individual nutritional goals of each patient. Thus, EFFORT does not provide evidence regarding single nutritional components or types of foods, but rather proves that the overall strategy of providing tailored nutritional support to reach the nutritional requirements during the acute phase of illness is beneficial for medical inpatients [30].

## 3. Nutritional Therapy

**Figure 1:**
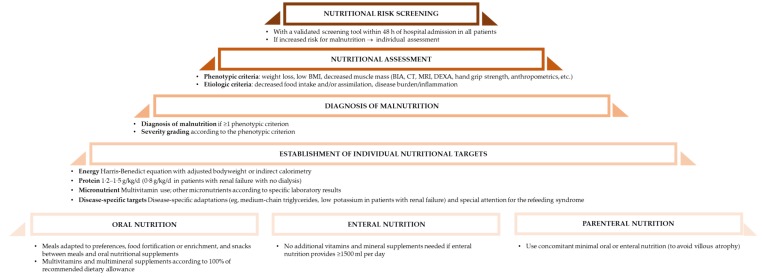
Nutritional algorithm, after [30].

Since there is growing evidence for the positive effect of nutritional therapy (Figure 1), proper definition and thus diagnosis are lacking. The Global Leadership Initiative on Malnutrition (GLIM) working group recently published an umbrella approach to diagnose malnutrition in various clinical settings [31]. This aims to early identify nutritional risk in polymorbid patients to individually treat them and improve their clinical outcome [31]. The GLIM working group supports a two-step process with screening and assessment, targeting a standardization of nutritional support [31].

There are differences of prognosis of nutritional status and nutritional strategy among the etiology of malnutrition; malnutrition with severe inflammation (e.g., severe infection, multiple trauma), with persistent inflammation due to chronic disease (e.g., cancer, congestive heart failure), malnutrition with minimum or no inflammation in chronic disease patients (e.g., short bowel syndrome, dysphagia after stroke and anorexia nervosa) and malnutrition due to simple starvation (e.g., poverty, dementia, lack of appropriate nutrition care in the hospital/facilities). Patients with severe inflammation are often difficult to recover their nutritional status by only nutritional support, so the goal of nutrition support is to minimize deterioration of nutritional status. On the other hand, nutritional status of the patients with no or minimum inflammation, or those after simple starvation, can be improved if appropriate nutrition support provided by the nutrition specialists.

### 3.1. Organization of Nutritional Support

Nutritional support in hospitals ideally relies on two main structures: A hospital nutrition steering committee and multidisciplinary nutritional support teams. The nutrition steering committee is working within the clinical governance framework (sets nutritional standards, protocols and guidelines) and has a direct access to hospital management. Members of the nutrition steering committee should be drawn from the management, and include senior representation from medical staff, catering, nursing, dietetics, pharmacy and other healthcare professionals [32,33]. A nutritional support team has an executive function within the hospital, implementing standards, protocols and guidelines in daily clinical practice. Such a team consisting of physicians, dieticians, nurses, and pharmacists, ensures and improves nutritional treatments quality and safety, and is continuously checking and optimizing procedures of nutritional management (see dedicated manuscript in this special issue). Interdisciplinary cooperation and good communication between healthcare professionals is important and mandatory to provide an individualized nutritional support to all patients who need it.

### 3.2. Screening and Assessment

The identification of malnutrition has been typically based on anthropometric, biochemical and physical parameters, among others. There is no universally accepted gold standard (best method) for the assessment of nutritional status [34,35]. Commonly used criteria include unintentional weight loss (percentage of body weight) in the past 3–6 months, low BMI, reduced muscle mass, reduced dietary intake in the past week, reduced absorptive capacity, and disease burden/inflammation [31].

Standardized procedures are needed in order to initiate a timely and adequate nutritional therapy [36]. Systematic screening for nutritional risk, followed by a comprehensive nutritional assessment should be implemented and lead to the development of a personalized nutritional care plan (see dedicated manuscript in this special issue: Reber et al., Nutritional screening and assessment) [13,33,37]. Nutrition screening is the first step in determining nutritional problems. Screening should rapidly and accurately identify individuals who should be referred to the nutrition specialist (e.g., dietitian, expert clinician) for a further assessment, where it would be possible to gather more information and determine if there truly is a nutrition problem, to understand the cause of the problem and to determine its severity. Nutritional screening should be done with validated screening tools, such as malnutrition universal screening (MUST), mini nutritional assessment short form (MNA-SF) and nutritional risk screening 2002 (NRS-2002), each of them having strength and limitations and may not be sufficiently validated in specific populations like polymorbid patients (see dedicated manuscript in this special issue: Reber et al., Nutritional screening and assessment) [38,39]. In general, nutritional assessment continues the data gathering process initiated in the screen. The types of data collected in nutritional assessment are often similar to data collected in the screening process but in more depth [40]. In summary, nutritional support should be initiated with a detailed nutritional care plan (appropriate monitoring procedures and clear guidelines for action e.g., food record charts or dietetic referral) in people who are at risk of malnutrition or manifestly malnourished according to the result of the nutritional risk screening and assessment results [41].

### 3.3. Nutritional Targets

Meeting the nutritional requirement is important to maintain or gain weight, muscle mass and function, to improve clinical outcomes, and reduce complications and rehospitalization rates.

Dietitians and medical staff trained in nutrition support should ensure that the patients’ dietary intake meets the individual energy, protein, fluid, electrolyte, mineral, micronutrients and fiber needs [33]. The activity levels and the underlying clinical condition (e.g., catabolism, pyrexia, gastrointestinal tolerance, potential metabolic instability), as well as the likely duration of nutrition support, should also be taken into account.

#### 3.3.1. Energy

Determining the patients’ energy requirements is a central point of nutritional assessment. The total energy expenditure consists of the resting energy expenditure, diet-induced thermogenesis and the energy expended during physical activity [13]. The gold standard to measure energy requirements is the indirect calorimetry, but when not possible, these may be calculated with published prediction equations adjusted for age sex, and weight (e.g., Harris-Benedict formula) in addition to activity and stress factors, or roughly estimated weight-based formulae (it has been suggested 27 kcal/kg/day for polymorbid older patients) [13]. In individuals with very low BMI, the higher value of 30 kcal/kg/d should slowly be targeted [13]. Reaching the energy targets too quickly and/or feeding at too high rates potentially lead to complications such as the refeeding syndrome or overfeeding [42].

#### 3.3.2. Proteins

The general protein requirement is 1 g/kg/d for the polymorbid medical inpatient population. In case of acute or chronic kidney failure, protein requirements may have to be reduced to 0.8–1 g/kg/d unless dialysis, but there are no specific recommendations for polymorbid patients suffering from kidney failure [13]. In case of dialysis, the protein requirements are the same as normal and there is a need for 20 g of proteins after the dialysis (dialytic loss). For older patients suffering acute or chronic disease, recommendations are 1.2–15. g/kg/d [43,44]. An individual target has to be defined for each patient, since other factors such as hypermetabolism can change protein requirements (e.g., major trauma and burns). Patients undergoing paracentesis need 10 g proteins per liter ascites.

#### 3.3.3. Micronutrients

In malnourished polymorbid inpatients, micronutrients requirements may be higher due to reduced food intake or due to higher needs (disease-depending). Micronutrients should be supplemented according to the recommended daily intake, and/or substituted if deficiencies are occurring [13]. The daily micronutrients requirements are considered to be covered in case of the amount of enteral nutrition is ≥1500 mL per day. Vitamins and trace elements should be provided to patients receiving parenteral nutrition since there are none in the nutritional solutions.

### 3.4. Nutritional Route

Oral nutrition should be the first-line choice of nutritional support, including meals adapted to the individual patient preferences, additional energy dense snacks (e.g., “homemade” milkshakes), food fortification with powdered carbohydrate and protein supplements, and the commercial oral nutritional supplements [13]. The use of the convenient commercial oral nutritional supplements have been shown to maintain the muscle mass, to lower the complication rate and hospital readmission rate after six months [13,45,46,47,48], but a combination of oral nutritional support strategies (using both traditional and commercially available products) also results in the improvement of important clinical outcomes [30].

If oral nutrition is not possible, safe or sufficient, enteral and eventually parenteral nutrition should be considered, whereas the enteral nutrition should be the preferred route due to lower risk of infectious and non-infectious complications [13]. The energy and protein intake should be assessed every 24–48 h and escalated after 5 days if the patient does not meet at least 75% of his/her requirements [49].

### 3.5. Nutrition for Specific Medical Conditions

Patients with renal failure need restrictions in potassium and phosphate. Patients with congestive heart failure may benefit from sodium and water restrictions in given cases.

There is weak evidence to support the use of specialized oral nutritional supplements or enteral nutrition formulas in polymorbid medical inpatients. Arginine, glutamine, and beta-hydroxy-beta-metylbuturate (HMB) may be used in patients suffering from pressure ulcers [13,50]. Arginine (semi-essential amino acid) is required for promotion of nitrogen balance, cell proliferation, T lymphocyte function and collagen accumulation. It also changes into nitric oxide, which is known for its vasodilatory and angiogenic properties. Glutamine (conditionally essential amino acid) plays a key role in the immune system, and its deficiency may significantly slow the healing process. HMB (metabolite of leucine, an essential amino acid) supplementation was associated with increased muscle mass accretion by inhibiting muscle proteolysis and modulating protein turnover. A mixture of soluble and insoluble fibers can be used in older polymorbid patients receiving enteral nutrition and suffering from diarrhea or constipation, which are frequent complications of enteral nutrition [13]. Special attention should then be paid to the hydration state as it may cause obstipation [51].

### 3.6. Timing

An early start of nutritional support (within 48 h after hospital admission) is recommended to maintain or improve patient’s nutritional and functional status, and prevent sarcopenia [13]. Even though the optimal length of the nutritional support is still unclear, it is usually recommended to treat beyond hospital discharge, as this continued nutritional intervention is known to increase patients’ quality of life and nutritional and functional status, and in the older (>65 years) polymorbid patients it results in lower mortality rates [13,52,53].

### 3.7. Monitoring

Experienced clinical teams with the relevant skills and training in nutritional monitoring should review the indications, route, risks, benefits and goals of nutrition support at regular intervals. This interval depends on the patient and the parameter that is being monitored (e.g., nutritional, anthropometric, biochemical, clinical condition), and may increase when the patient’s condition is stable under nutritional treatment. For example, nutrient intake from oral, enteral or parenteral nutrition (including any change in conditions that are affecting food intake) is recommended to be monitored daily initially, reducing to twice weekly when stable [33]. Similarly, laboratory parameters (e.g., sodium, potassium, urea, creatinine) should be monitored more frequently initially, i.e., at baseline and daily until stable, and then reduced to 1 or 2 times a week. In addition to the nutritional parameters used to monitor responses to nutritional support, functional indices (e.g., handgrip strength) should be used regularly to asses other clinical outcomes (i.e., survival, quality of life) in polymorbid medical inpatients.

## 4. Barriers to the Adequate Provision of Nutritional Support

The awareness about malnutrition must be raised primarily among hospital medical staff, where nutritional support is not seen as being part of the medical treatment. Malnutrition is often unrecognized as a diagnosis and is, subsequently, being under-reported and not being treated. Clearly defined protocols and responsibilities are needed to address this hidden problem, starting with a systematic nutritional risk screening and nutritional assessment for all patients on admission to hospital. Malnutrition should become a topic in the education and training of medical staff. At the same time, because the causes of malnutrition are often multifactorial (from depression and lack of appetite to inability to self-feed), communication and collaboration should be improved between the different members of the multidisciplinary team and between the team and the patient, in order to efficiently address those causes [16]. A recently published study showed that 78% of the hospitals and 22% of the nursing homes in Switzerland have a nutritional steering committee, and 92% and 14%, respectively, have dieticians available. Around 80% of the hospitals and nursing homes did not implement a systematic nutritional risk screening procedure, resulting in around 60% of the patients being screened at least at one time point (in hospitals and nursing homes); 25% of the hospital do screen the patients as they feel a problem [54]. Further, 56% of the institutions monitor the food intake of their patients and 50% monitor and document the nutritional status [54]. The nutritionDay survey 2018, a survey on nutritional care conducted worldwide (https://www.nutritionday.org/en/network/imprint/index.html; Table 1) showed that 76.4% of the participating centers had NST available on site, and that 61.6% of the participating centers use a specific screening tool to screen the patients at hospital admission.

Individual factors such as patients’ symptoms, disease severity (e.g., causing dysphagia and loss of appetite), mood and orientation, impaired functional and cognitive status, social environment (e.g., isolation, loneliness, lack of family support) may all significantly reduce the food (and nutrient) intake of hospitalized patients and contribute for the deterioration of their nutritional status. Polypharmacy, especially in polymorbid patients, may further influence their nutritional status, causing drug–drug and drug–nutrients interactions as well as gastrointestinal symptoms. This calls for the need of frequent monitoring of the nutritional status of the hospitalized patient (e.g., weekly monitoring of the body weight). For those patients receiving nutritional support, regular visits by the dietitian allow the patient to receive the encouragement to comply with the nutritional care plan, and the adaptation of this plan to the changing needs of the patient [55].

## 5. Conclusions and Outlook

Nutritional support has been shown to be a highly effective treatment option to prevent and/or treat malnutrition, decreasing morbidity and mortality rates. Nutritional interventions deserve as much attention as any other therapeutic interventions, and clinicians should aim to maximize their efficacy and minimize side effects. Further studies should also investigate the cost-effectiveness of nutritional interventions in medically ill patients. It will be equally important to determine which medical inpatients have the most benefit from nutritional support interventions through metabolomics and microbiome research, walking towards an evidence-based personalized nutrition approach.

## Figures and Tables

**Table 1:** National reports from the nutritionDay 2018, available from https://www.nutritionday.org/en/about-nday/national-reports/index.html.

Country	Number of Participating Centers	Percentage of Hospitals with NST Present on Site	Percentage of Screening at Hospital Admission
Australia	1	100	100
Austria	4	100	45.5
Belgium	52	88	77.3
Brazil	12	100	80.0
Bulgaria	1	66.7	0
China	9	100	56.5
Colombia	35	69.1	56.6
Croatia	3	55.6	20.0
Germany	36	60.9	49.5
Greece	3	100	0
India	13	7.7	50.1
Japan	9	100	50
Netherlands	1	100	0
Poland	4	0	60
Portugal	4	100	66.7
Singapore	1	0	82.6
Spain	5	100	50.0
Sweden	2	100	100
Switzerland	1	66.7	16.7
Thailand	1	100	66.7
United States of America	16	62.5	96.0
